# Short-duration hypothermia completed prior to reperfusion prevents intracranial pressure elevation following ischaemic stroke in rats

**DOI:** 10.1038/s41598-021-01838-7

**Published:** 2021-11-16

**Authors:** Daniel Omileke, Sara Azarpeykan, Steven W. Bothwell, Debbie Pepperall, Daniel J. Beard, Kirsten Coupland, Adjanie Patabendige, Neil J. Spratt

**Affiliations:** 1 The School of Biomedical Sciences and Pharmacy, University of Newcastle, Callaghan, NSW Australia; 2 Hunter Medical Research Institute, New Lambton, NSW Australia; 3 Institute of Infection, Veterinary & Ecological Sciences, University of Liverpool, Liverpool, UK; 4 Department of Neurology, John Hunter Hospital, Hunter New England Local Health District, New Lambton, NSW Australia

**Keywords:** Neurological disorders, Neuro-vascular interactions

## Abstract

Reperfusion therapies re-establish blood flow after arterial occlusion and improve outcome for ischaemic stroke patients. Intracranial pressure (ICP) elevation occurs 18–24 h after experimental stroke. This elevation is prevented by short-duration hypothermia spanning the time of reperfusion. We aimed to determine whether hypothermia-rewarming completed prior to reperfusion, also prevents ICP elevation 24 h post-stroke. Transient middle cerebral artery occlusion was performed on male outbred Wistar rats. Sixty-minute hypothermia to 33 °C, followed by rewarming was induced prior to reperfusion in one group, and after reperfusion in another group. Normothermia controls received identical anaesthesia protocols. ΔICP from pre-stroke to 24 h post-stroke was measured, and infarct volumes were calculated. Rewarming pre-reperfusion prevented ICP elevation (ΔICP = 0.3 ± 3.9 mmHg vs. normothermia ΔICP = 5.2 ± 2.1 mmHg, *p* = 0.02) and reduced infarct volume (pre-reperfusion = 78.6 ± 23.7 mm^3^ vs. normothermia = 125.1 ± 44.3 mm^3^, *p* = 0.04) 24 h post-stroke. There were no significant differences in ΔICP or infarct volumes between hypothermia groups rewarmed pre- or post-reperfusion. Hypothermia during reperfusion is not necessary for prevention of ICP rise or infarct volume reduction. Short-duration hypothermia may be an applicable early treatment strategy for stroke patients prior to- during-, and after reperfusion therapy.

## Introduction

Reperfusion is necessary for the maintenance of tissue integrity in the ischaemic penumbra. Without reperfusion, any neuroprotective treatment measure is unlikely to show efficacy in stroke patients^1^. Previous studies by our group have demonstrated a dramatic increase in intracranial pressure (ICP) after transient middle cerebral artery (MCA) occlusion in rats^2–6^. We have also demonstrated that short-duration hypothermia prevents this ICP elevation^4–6^. However, in studies to date, hypothermia treatment has always spanned the reperfusion phase of the transient MCA occlusion model.

Reperfusion marks a time of major activity in the ischaemic brain. Reperfusion after ischaemic stroke is defined as the restoration of blood supply to the damaged cerebral tissue. Successful reperfusion significantly improves functional outcome in stroke patients and increases the likelihood of disability free survival^7–9^. However, reperfusion therapies carry some risks and in some patients, the rapid restoration of blood flow increases the risk of symptomatic intracerebral haemorrhage^10^, and may induce secondary damage to the penumbral region in what is known as ischaemic-reperfusion injury (R/I)^11,12^. A main contributing pathological mechanism to R/I is the generation of reactive oxygen species (ROS). R/I can become a more serious problem when reperfusion therapy is performed outside of the critical time window for treatment. The sudden introduction of blood flow into the already compromised ischaemic brain tissue may overwhelm the exhausted endogenous antioxidant systems and damaged vascular endothelia, resulting in cerebral oedema and extravasation of blood cells^13^. The infiltration of leukocytes into the ischaemic brain during reperfusion, as well as the activation of resident microglia are known to play a role in R/I induced inflammation but may also cause further ROS activation^14^, thereby creating a vicious cycle of secondary damage^15^. Hypothermia has also been shown to be protective against these R/I processes described above^16^.

Given what we know about hypothermia and ICP elevation, it is possible that cooling during the reperfusion phase may be important for ICP rise prevention post-stroke. While hypothermia treatment has been shown to reduce infarct volume independent of reperfusion in animal studies^17^, these studies did not examine ICP so it is unclear whether hypothermia during reperfusion is necessary for prevention of ICP rise. To assess the importance of cooling during reperfusion, we aimed to determine whether hypothermia-rewarming completion prior to reperfusion onset prevents ICP elevation 24 h following ischaemic stroke, compared to normothermia control rats. For direct comparison with our previous work, we included a second hypothermia group that were cooled during the reperfusion phase i.e., rewarmed after reperfusion. This group acted as an active comparator, on the basis that we have abundant evidence demonstrating the benefits of cooling during the reperfusion phase on ICP^4–6^. We also aimed to determine whether hypothermia-rewarming pre-reperfusion would reduce infarct volume and neurological deficit post-stroke.

## Results

A total of 5 animals were excluded from this study. One was excluded due to lack of sufficient drop in perfusion units (> 50% drop from baseline in MCA territory) at occlusion (pre-randomisation); 2 were excluded due to sudden cessation of breathing after hypothermia initiation (rewarmed pre-reperfusion); 2 due to subarachnoid haemorrhage, confirmed at post-mortem (normothermia).

**Figure 1:**
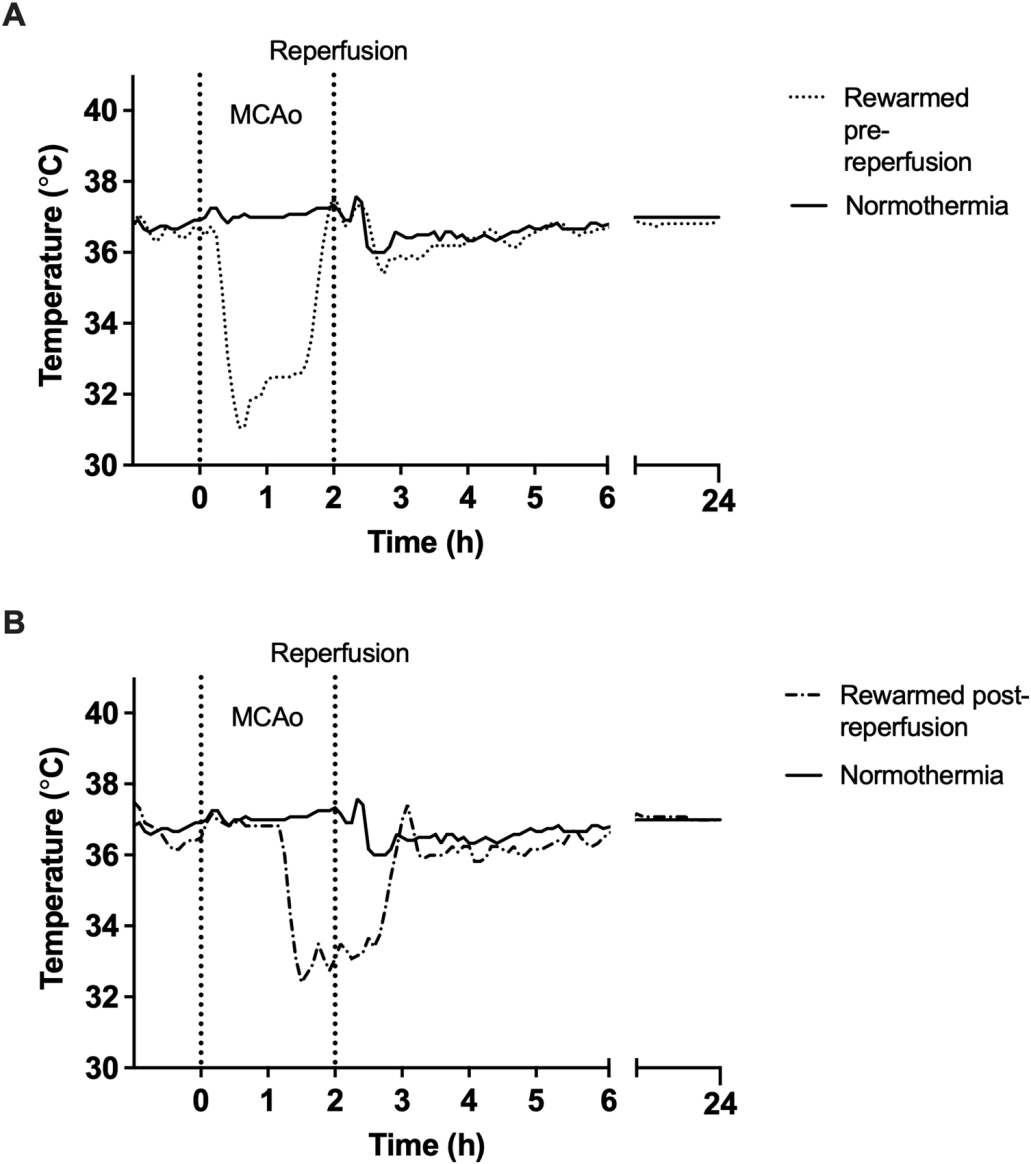
Core body temperature data. (**A**) rewarmed pre-reperfusion and normothermia. Hypothermia was initiated 15 min post-MCAo. After target temperature was reached, hypothermia was maintained for 1 h before rewarming, and prior to reperfusion at 2 h post-stroke. (**B**) rewarmed post-reperfusion and normothermia. Hypothermia was initiated 75 min post*-*MCAo. After target temperature was reached, hypothermia was maintained for 1 h before rewarming was initiated*.* Reperfusion had already occurred at the point of rewarming initiation in this group.

Mean temperature was 32.7 ± 1.6 °C during treatment in the hypothermia groups (n = 12). Mean temperature was 37.0 ± 0.6 °C during the equivalent period in the normothermia group (n = 6) (Fig. 1).

**Figure 2:**
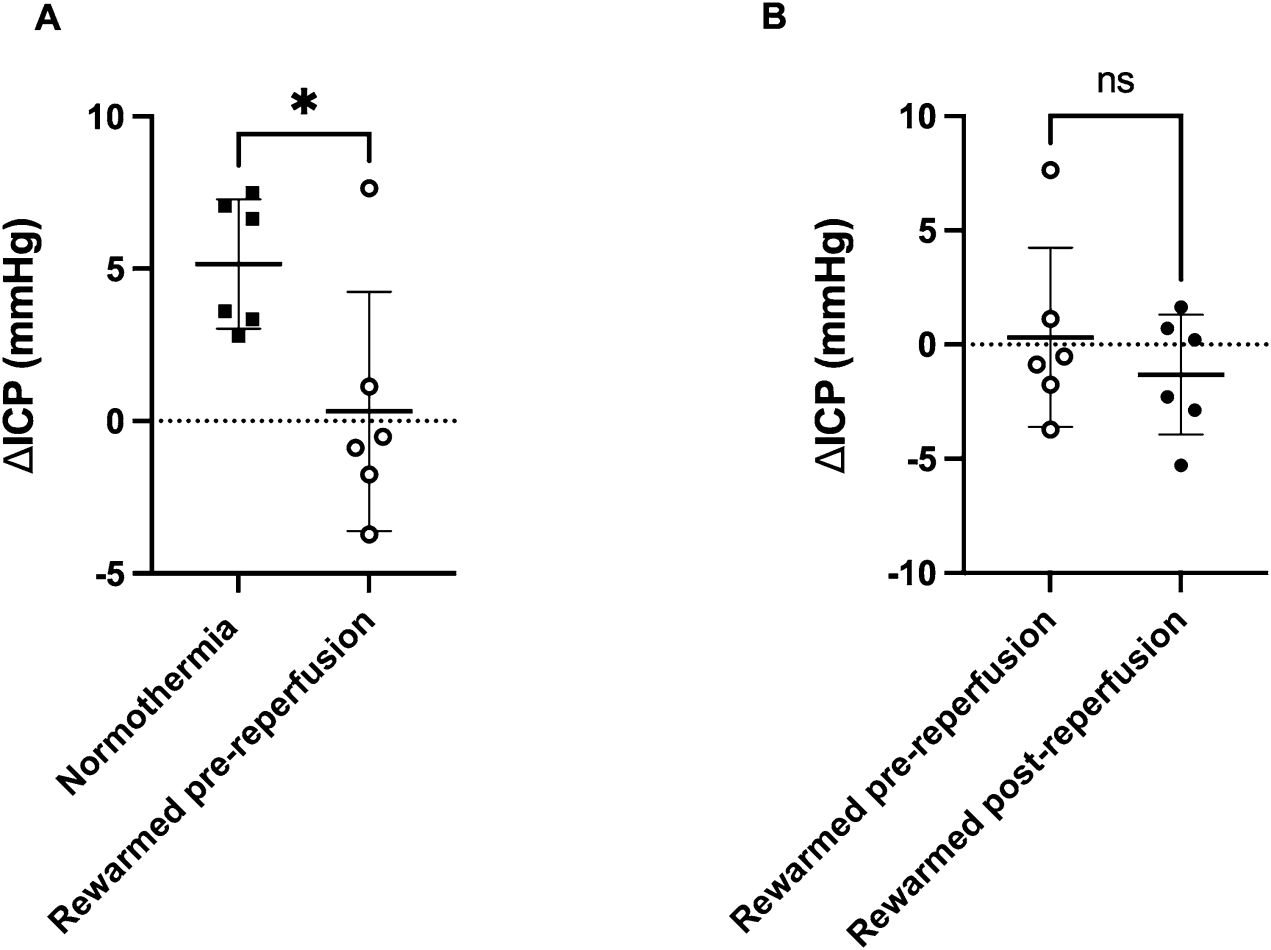
**(A**) Change in ICP from baseline to 24 h (∆ICP) between rewarmed pre-reperfusion and normothermia groups 24 h post-stroke. (**B**) ∆ICP between rewarmed pre-reperfusion and rewarmed post-reperfusion hypothermia groups 24 h post-stroke. *p < 0.05. *NS* not significant.

ICP values were 3.58 ± 1.23 mmHg and 3.12 ± 2.52 mmHg at baseline (non-significant), and 3.89 ± 3.50 mmHg and 8.34 ± 2.99 mmHg at 24 h (*p* = 0.03) in the rewarmed pre-reperfusion and normothermia groups, respectively. ∆ICP was significantly lower in the rewarmed pre-reperfusion group versus the normothermia group (0.3 ± 3.9 mmHg vs. 5.2 ± 2.1 mmHg, *p* = 0.02: Fig. 2A). In the rewarmed post-reperfusion group, ICP values were 4.54 ± 1.56 mmHg at baseline and 3.22 ± 1.59 mmHg at 24 h, which were not significantly different when compared with rewarmed pre-reperfusion ICP. There was no significant difference in ∆ICP between the rewarmed pre-reperfusion and rewarmed post-reperfusion groups (0.3 ± 3.9 mmHg vs. − 1.3 ± 2.6 mmHg, *p* = 0.42: Fig. 2B).

**Figure 3:**
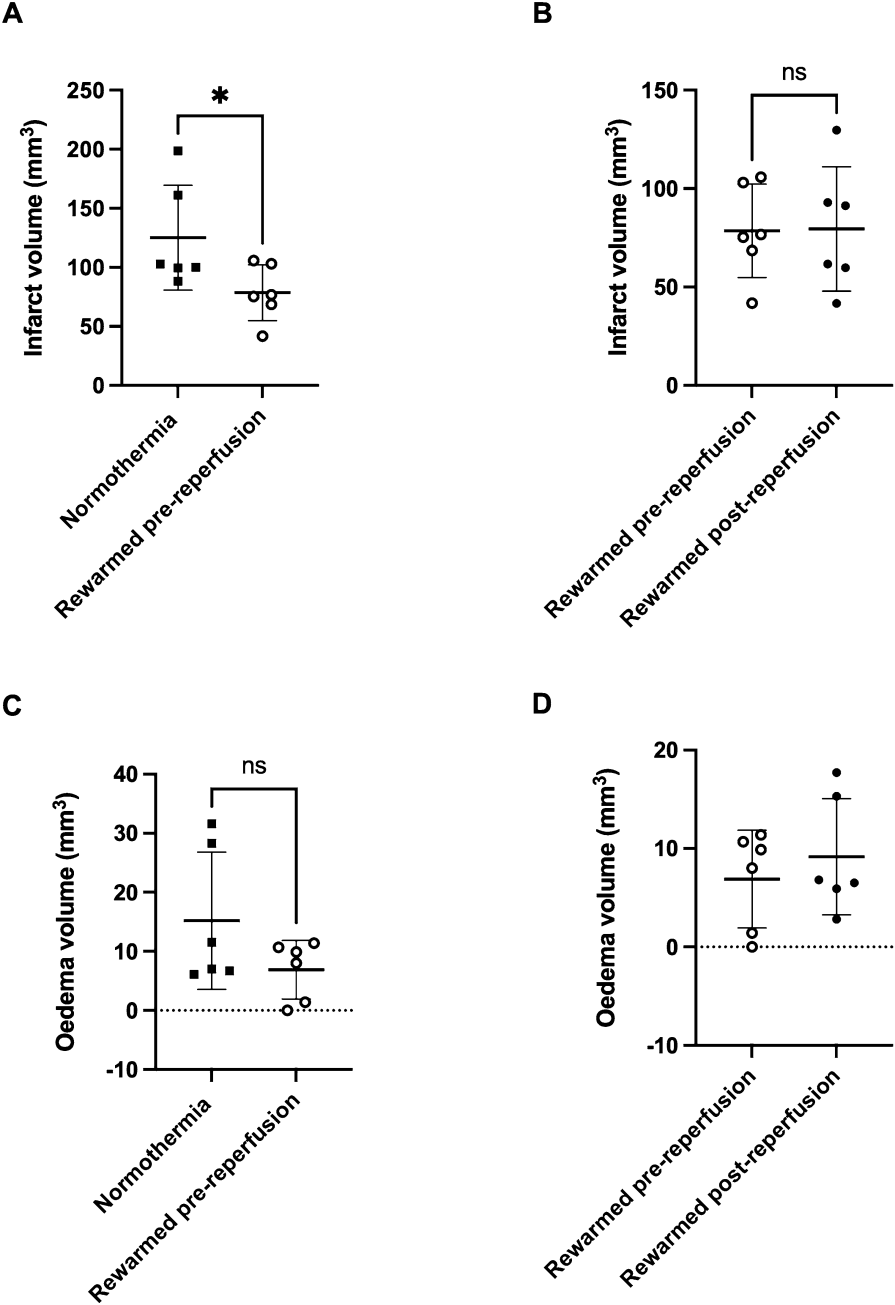
Infarct and oedema volume from H&E staining. (**A**, **C**) Rewarmed pre-reperfusion and normothermia, and (**B**, **D**) rewarmed pre-reperfusion and rewarmed post-reperfusion groups. *p < 0.05. *NS* not significant.

Infarct volume 24 h post-stroke was significantly smaller in the rewarmed pre-reperfusion group versus the normothermia group (78.6 ± 23.7 mm^3^ vs. 125.1 ± 44.3 mm^3^, *p* = 0.04: Fig. 3A). There was no significant difference in infarct volume between the rewarmed pre-reperfusion and rewarmed post-reperfusion groups (78.6 ± 23.7 mm^3^ vs. 79.5 ± 31.5 mm^3^, *p* = 0.55: Fig. 3B). There was no significant difference in oedema volume between the rewarmed pre-reperfusion and normothermia groups (6.9 ± 4.9 mm^3^ vs. 15.2 ± 11.6 mm^3^, *p* = 0.14: Fig. 3C). Rewarmed pre-reperfusion and rewarmed post-reperfusion data are shown in Fig. 3D. Statistical analysis was not performed on this dataset because there was no significant difference between the rewarmed pre-reperfusion and normothermia groups (as per our pre-specified analysis plan).

**Figure 4:**
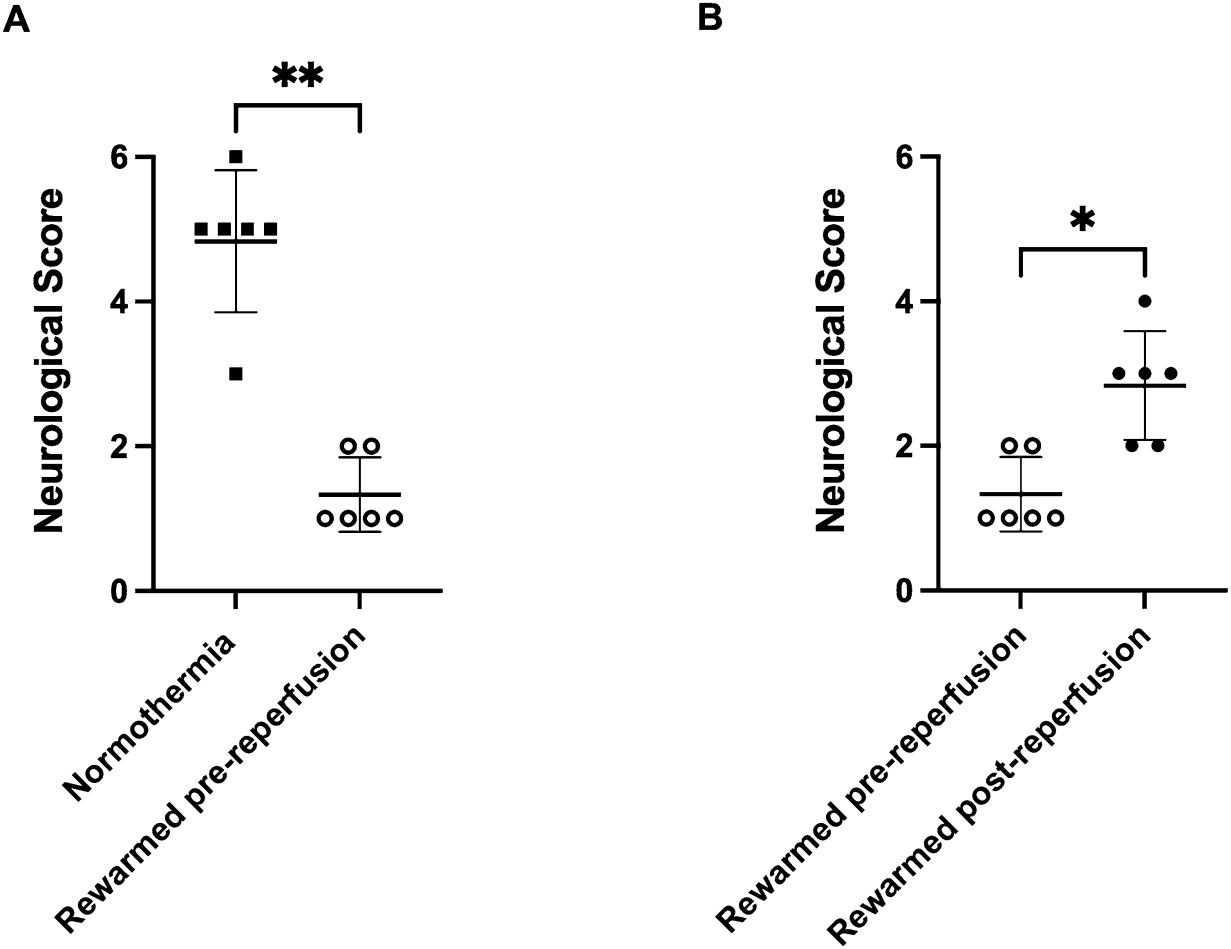
Neurological deficit scores in (**A**) rewarmed pre-reperfusion and normothermia and (**B**) rewarmed pre-reperfusion and rewarmed post-reperfusion animals. *p < 0.05, **p < 0.01.

Neurological deficit scores were significantly lower in the rewarmed pre-reperfusion group when compared to the normthermia group (1.3 ± 0.5 vs. 4.8 ± 1.0, *p* = 0.002: Fig. 4A). There was also a significant difference in neurological deficit scores amongst the two hypothermia groups. Rewarmed pre-reperfusion animals had lower scores compared to the rewarmed post-reperfusion animals (1.3 ± 0.5 vs. 2.8 ± 0.7, *p* = 0.01: Fig. 4B).

## Discussion

In this study, we have shown that 60 min hypothermia to 33 °C with rewarming pre-reperfusion, prevented ICP elevation, reduced infarct volume, and reduced neurological deficit 24 h post-stroke compared to normothermia controls. We have also shown that there was no significant difference between rewarming before reperfusion and rewarming after reperfusion in terms of ICP rise prevention or infarct volume reduction at the same time point.

These results further our understanding of the protective effects of short-duration hypothermia by providing evidence of cerebroprotection even when hypothermia treatment is completed prior to reperfusion. In all our previous studies, reperfusion occurred during the cooling interval^4–6^. This was an important timepoint to assess as it is necessary to ensure that hypothermia is effective at preventing ICP elevation in reperfused brain. Cooling during the reperfusion phase also has clinical relevance as reperfusion therapies are the gold standard treatment for ischaemic stroke^18^, therefore having a treatment strategy that can be used as an adjunct to reperfusion therapies is advantageous. The results from the present study indicate that hypothermia may be suitable as a potential early treatment strategy following a stroke, prior to reperfusion therapy. Here, animals in the rewarmed pre-reperfusion group reached target temperature 30 min following occlusion and were only maintained at target for 1 h, yet we saw significant ICP rise prevention and infarct volume reduction as were previously reported when animals are cooled during the reperfusion period^4,19^. This provides evidence that early administration of short-duration hypothermia may be beneficial to stroke patients, independent of whether it straddles the reperfusion interval, in those receiving reperfusion therapies.

Our results also suggest that the earlier onset of cooling may enhance the benefits of hypothermia. Animals that were rewarmed pre-reperfusion had significantly lower neurological deficits than animals rewarmed post-reperfusion. This result is interesting when we consider that infarct volumes did not differ significantly between treatment groups, yet functional outcomes did. Functional outcomes more closely align with the clinical situation, i.e. the modified Rankin scale^20^. These results suggest that pre-reperfusion hypothermia-rewarming may be of greater benefit to stroke patients in terms of functional outcome. These neurological deficit results could likely be explained by the timing of hypothermia initiated in both groups. In order to keep the duration of both MCA occlusion and hypothermia identical between groups, hypothermia was initiated 15 min after MCAo in the rewarmed pre-reperfusion group but was initiated 75 min after MCAo in the rewarmed post-reperfusion group. Perhaps an earlier treatment strategy in the absence of reperfusion is responsible for better neurological outcome^21^. It is possible that R/I mechanisms may have advanced too far in reperfused brain for hypothermia to elicit its maximal therapeutic benefit. However, interpretation requires a little caution since ICP, and particularly, infarct volume , were not significantly different between treatment groups.

We have shown that cooling during reperfusion is not critical for ICP rise prevention post-stroke. This finding has important clinical implications. Pre-reperfusion hypothermia has been investigated extensively in the literature in studies of cardiac injury in animals^22^, but also in clinical investigations^23,24^. Pre-reperfusion hypothermia has also been examined in the context of stroke in animal studies^17,25^. Although these studies initiate hypothermia prior to reperfusion, the cooling period is maintained long after reperfusion has occurred, therefore these studies resemble the rewarmed post-reperfusion group in this present study. It is important to note that this study utilised very short durations at target temperature. The 60 min at target temperature is half the cooling time of previous pre-clinical investigations^4–6^, and 1/24th or less of the time of any of the human studies^26^. Long duration hypothermia in clinical trials have reported major logistic and tolerability issues^27–29^. The results obtained in this study may therefore make a significant difference to our approach to therapeutic hypothermia in stroke patients, as hypothermia may only need to be maintained for 60 min to show benefit.

This study demonstrates the previously reported consistency of ICP and infarct data in response to hypothermia treatment^19^. However, there are a number of limitations that must be noted. First, there was an unavoidable difference in hypothermia start time between the two treatment groups. It was necessary to match the groups for ischaemic duration and cooling duration, which therefore meant that cooling onset time could not be matched in order to investigate the importance of cooling during reperfusion. Second, this study was not powered for behavioural analyses. This study was powered for a primary outcome of change in ICP, not for neurological deficit scores, which typically require much larger sample sizes^30^. Lastly, we did not assess the deeper molecular mechanisms relating to the potential effects of hypothermia treatment on R/I events such as ROS generation or inflammation. Such investigations were beyond the scope of this current study, which was focused on exploring the importance of reperfusion on ICP elevation. Further work to investigate the molecular mechanisms will be necessary to elucidate potential differences between animals rewarmed pre- and post-reperfusion.

In conclusion, our results suggest that very short duration hypothermia-rewarming prior to reperfusion should be investigated for potential benefit to stroke patients. Reperfusion status can vary in patients and it would therefore be logistically challenging to implement hypothermia during the reperfusion period for all stroke patients. The results presented here indicate that therapeutic hypothermia might be a broadly applicable, early treatment option in stroke. Pre-reperfusion hypothermia-rewarming could be administered to stroke patients in the early acute phase, and irrespective of their reperfusion status at the time of treatment. This may enhance the clinical feasibility of therapeutic hypothermia in stroke patients.

## Materials and methods

### Animals and experimental protocol

Adult male outbred Wistar rats aged 11–12 weeks old (n = 23, Animal Services Unit, University of Newcastle) weighing 280–320 g were used for this study. Animals were housed under standard conditions in a 12 h light–dark cycle with unlimited access to food and water. All experimental procedures were in accordance with the Australian code of Practice for the Care and Use of Animals for Scientific Purposes and were approved by the Animal Care and Ethics Committee of the University of Newcastle (A-2020-003). This study was reported in accordance with the ARRIVE guidelines^31^.

Rats were initially anaesthetised in 5% isoflurane in 50:50 N_2_:O_2_ in an induction chamber. Anaesthesia was maintained with 2–2.5% isoflurane in the same gas mix and delivered via a custom, low dead space face mask with cross flow of gases. Incision sites were shaved, cleaned and injected subcutaneously (s.c.) with 2 mg/kg 0.05% Bupivacaine (Pfizer, Sydney, Australia). Body temperature was regulated throughout the surgery with a rectal temperature thermocouple (RET-2, Physitemp Instruments Inc, Clifton, New Jersey, USA). The femoral artery was cannulated with a catheter consisting of 1 and 2 French silicone tubing for continuous monitoring of arterial blood pressure and heart rate. Prior to recovery, rectal paracetamol (250 mg/kg, GlaxoSmithKline, Brentford, UK) was administered for overnight pain relief. Animals were also injected with saline (2 × 1.5 ml, s.c.) to prevent dehydration, and were returned to their cages with free access to softened laboratory chow and water.

### Implantation of datalogger device

Datalogger implantation was performed for accurate and continuous monitoring of core body temperature. Implantation was performed according to previously described methods^32^. A 2 cm longitudinal incision was made along the right abdominal region, proximal to the right thigh. The incision was made deep enough to expose the space at the ventral thigh crease. Haemostats and forceps were used to create a pocket under the skin that was large enough to hold the device. The temperature monitoring datalogger (Maxim, San Jose, USA) was inserted into the pocket and secured by closing the muscle and skin with 4-0 nylon sutures. Temperature measurements were logged every minute over the 24 h period. For analysis, 5 min recording intervals were chosen.

### Intracranial pressure and laser Doppler measurement

Cranial surgery was performed according to previously described methods^33^. To summarise, the ICP probe (OpSens Fibre Optic Pressure Sensors, Canada) was inserted epidurally into a saline filled, polyether ether ketone (PEEK) screw (Bregma 2 mm posterior and 2 mm lateral) in the left parietal bone. Tissue perfusion in the territory supplied by the right middle cerebral artery was monitored during middle cerebral artery occlusion (MCAo) and reperfusion using laser Doppler flowmetry (LDF). The LDF probe (Moor Instruments, UK) was inserted into a second hollow PEEK screw (Bregma 2 mm posterior and 5 mm lateral) in the right parietal bone. For ICP and LDF recordings, the screws were secured with dental cement and an airtight seal was created around each probe using a caulking material (Silagum, Gunz Dental, Germany). Correct placement of the ICP probe was confirmed by a response to abdominal compression which was observed on both ICP and arterial blood pressure waveforms. ICP was monitored at pre-stroke baseline and again at 24 h post-stroke. To account for minor variation between the baseline ICP of the 3 experimental groups, change in ICP from baseline to 24 h (∆ICP) was used for all ICP analyses.

### Middle cerebral artery occlusion

Transient MCAo was carried out according to our established protocol^34,35^. To summarise, a 6 cm length of monofilament nylon suture with a silicone tip (3 mm length × 0.38 mm O.D silicone) was inserted through the ligated right external carotid artery into the right internal carotid artery. The filament was advanced 20 mm through the internal carotid artery, avoiding the pterygopalatine artery, until resistance was felt, and a drop in perfusion units (> 50% drop from baseline) on the LDF was observed which indicated that the middle cerebral artery has been occluded. At 2 h post-occlusion, reperfusion was achieved by retracting the monofilament through the internal carotid artery approximately 18 mm until the silicone tip was visible in the external carotid artery stump.

### Hypothermia treatment

**Figure 5:**
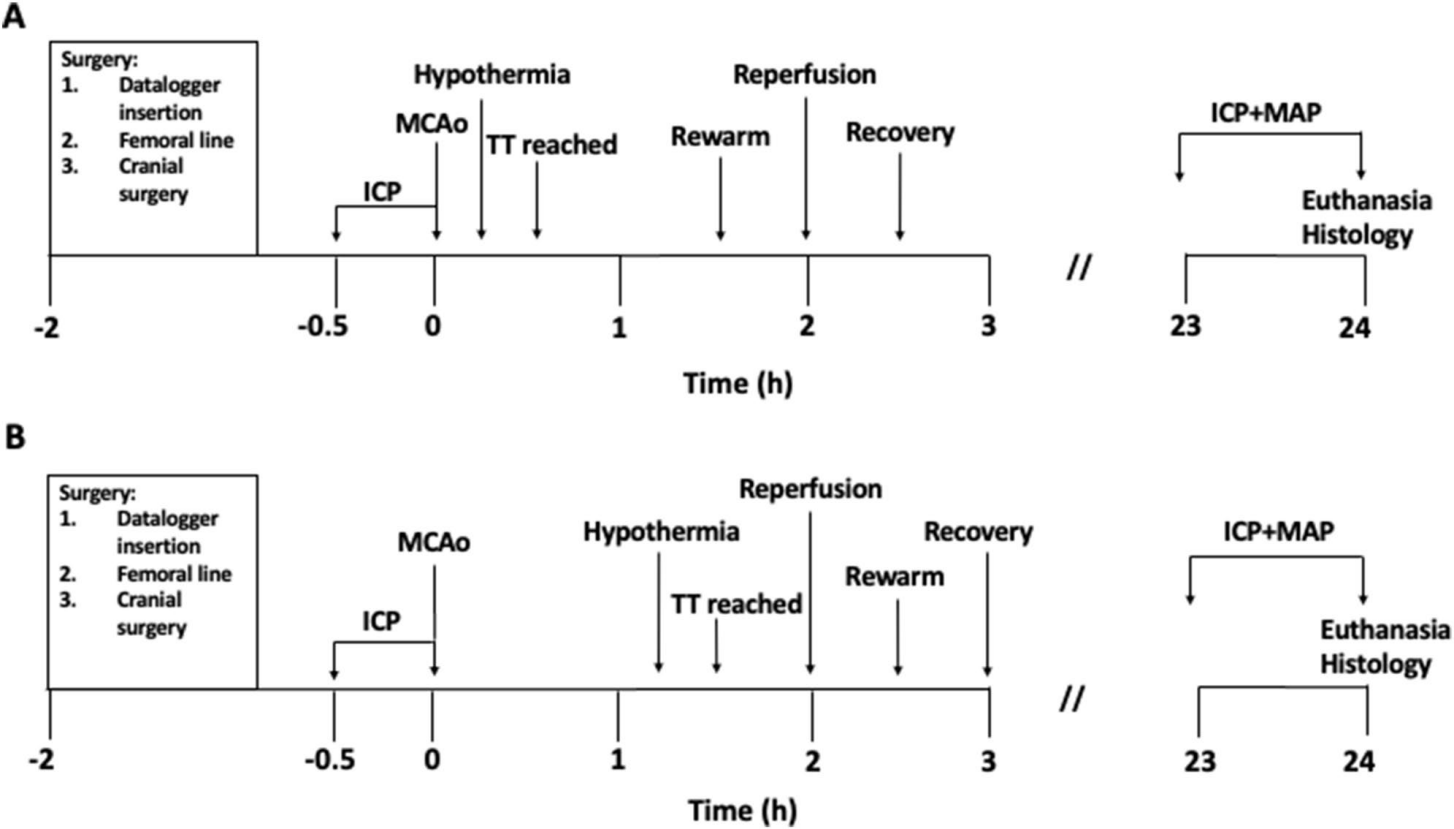
Experimental timeline of hypothermia treatment groups. (**A**) Shows the timeline for the rewarmed pre-reperfusion group, where hypothermia-rewarming is completed prior to reperfusing the rat at 2 h post-stroke. (**B**) Shows the timeline for the rewarmed post-reperfusion group, where hypothermia-rewarming is completed after reperfusion. *ICP* intracranial pressure, *MCAo* middle cerebral artery occlusion, *TT* target temperature, *MAP* mean arterial blood pressure.

Immediately after MCAo, animals were randomised by sealed envelope to rewarmed pre-reperfusion, rewarmed post-reperfusion or a normothermia group. Target temperature in both hypothermia groups was 33 °C and was achieved by the application of fans and ethanol spray using previously described methods^4,32^. In the rewarmed pre-reperfusion group, hypothermia treatment began 15 min after MCAo. Once 33 °C was reached, target temperature was maintained for 60 min. At the end of the 60 min cooling period, animals were rewarmed back to 37 °C, before reperfusion had occurred (Fig. 5A). In the rewarmed post-reperfusion group, hypothermia treatment began 75 min after MCAo. Once 33 °C was reached, target temperature was maintained for 60 min. At the end of the cooling period, animals were rewarmed back to 37 °C. In this rewarmed post-reperfusion group, reperfusion had already occurred when animals were being rewarmed (Fig. 5B). After treatment or normothermia, animals were placed in a cage half over a warming pad (Passwell, South Australia) to aid in thermoregulation and prevent post-operative hypothermia. Animals in the normothermia group were maintained at 37 °C for the duration of the surgery.

### Neurological tests

Prior to post-stroke ICP monitoring at 24 h, animals were tested for stroke-induced neurological deficits. The neurological deficit score was composed of the forelimb flexion, torso twist, and lateral push tests. Each test was ranked from 0 to 2, and a total score was given out of 6 (higher scores indicated greater neurological deficit)^36^.

### Histological analysis and infarct volume measurement

Animals were euthanised 24 h post stroke onset. They were transcardially perfused with saline and their brains were removed and sectioned into 6 coronal slices using a rat brain matrix, each of 2 mm thickness. Triphenyltetrazolium chloride (TTC) (Sigma-Aldrich, Missouri, USA) staining was performed to confirm the presence of ischaemic stroke by identification of infarcted tissue (white area). The slices from each brain were incubated for 12 min at 37 °C in 2% TTC. TTC was used for early confirmation of infarct, however, these same tissue slices were then fixed, processed, paraffin embedded and cut into 5 µm coronal sections for haematoxylin and eosin (H&E) staining. H&E staining was used for infarct volume quantification. Images were scanned using a high-resolution scanner (Aperio, Vista, CA, USA) and analysed by an investigator blinded to treatment allocation. Infarct (corrected for oedema) was calculated (Aperio ImageScope) by subtracting the measured interhemispheric volume difference from the measured infarct volume for each side. Infarct volumes were corrected for oedema by applying the formula: corrected infarct volume (mm^3^) = infarct volume × (contralateral volume/ipsilateral volume). Oedema was calculated by infarct volume minus corrected infarct volume^5^.

### Exclusion criteria and statistical analysis

Subarachnoid haemorrhage, equipment malfunction and absence of > 50% LDF drop at occlusion were pre-specified exclusion criteria.

A sample size calculation was performed using pilot and previous data^4,5^ (G*Power version 3.1) which indicated that 3 animals per group were required to detect a 6 mmHg difference in ∆ICP between the rewarmed pre-reperfusion and normothermia groups, with standard settings of alpha 0.05, power 0.8. However, we used a minimum sample size of 6 animals per group to allow for outlier effects. Statistical analyses were performed using GraphPad Prism version 9.0.1. Data were analysed for normal distribution using the Shapiro–Wilk normality test. According to the prespecified analysis plan, a primary one-way analysis with two levels (unpaired t-test) was conducted to examine if there was a statistical difference between the rewarmed pre-reperfusion and normothermia groups using ∆ICP as the primary endpoint. If a significance difference was found, a subsequent one-way, two-level analysis was conducted between the rewarmed pre-reperfusion and the rewarmed post-reperfusion (active comparator) group. The same protocol was used for infarct volume and oedema analyses. For non-normally distributed data (neurological deficit scores), the Mann–Whitney U test was conducted using the same protocol as above. Statistical significance was accepted at *p* < 0.05. Data are presented as mean ± SD unless otherwise stated.
